# Supplementary material for: 3D reconstruction from cryo-EM projection images using two spherical embeddings
Source: Commun Biol. 2022 Apr 4;5:304. doi: 10.1038/s42003-022-03255-6 (PMC8979997; doi:10.1038/s42003-022-03255-6)
Supplement: Supplementary file 2 — Reporting Summary [file 42003_2022_3255_MOESM2_ESM.pdf]

nature research

Corresponding author(s): Yonggang Lu

Last updated by author(s): Jan 31, 2022

Reporting Summary

Nature Research wishes to improve the reproducibility of the work that we publish. This form provides structure for consistency and transparency in reporting. For further information on Nature Research policies, see our Editorial Policies and the Editorial Policy Checklist.

Statistics

For all statistical analyses, confirm that the following items are present in the figure legend, table legend, main text, or Methods section.

n/a	Confirmed	
☐	☒	The exact sample size (*n*) for each experimental group/condition, given as a discrete number and unit of measurement
☒	☐	A statement on whether measurements were taken from distinct samples or whether the same sample was measured repeatedly
☒	☐	The statistical test(s) used AND whether they are one- or two-sided<br>*Only common tests should be described solely by name; describe more complex techniques in the Methods section.*
☒	☐	A description of all covariates tested
☒	☐	A description of any assumptions or corrections, such as tests of normality and adjustment for multiple comparisons
☐	☒	A full description of the statistical parameters including central tendency (e.g. means) or other basic estimates (e.g. regression coefficient) AND variation (e.g. standard deviation) or associated estimates of uncertainty (e.g. confidence intervals)
☒	☐	For null hypothesis testing, the test statistic (e.g. *F*, *t*, *r*) with confidence intervals, effect sizes, degrees of freedom and *P* value noted<br>*Give P values as exact values whenever suitable.*
☒	☐	For Bayesian analysis, information on the choice of priors and Markov chain Monte Carlo settings
☒	☐	For hierarchical and complex designs, identification of the appropriate level for tests and full reporting of outcomes
☒	☐	Estimates of effect sizes (e.g. Cohen's *d*, Pearson's *r*), indicating how they were calculated

Our web collection on statistics for biologists contains articles on many of the points above.

Software and code

Policy information about availability of computer code

Data collection	RELION-2 was used to generate the 2D class averages for the real data.
Data analysis	The following tools were used for data analysis: EMAN 2.1, RELION-2 , ASPIRE 0.14 (http://spr.math.princeton.edu), 3D reconstruction using spherical embedding (https://github.com/yluATlzu/3DReconstruction_SE), Chimera 1.13.

For manuscripts utilizing custom algorithms or software that are central to the research but not yet described in published literature, software must be made available to editors and reviewers. We strongly encourage code deposition in a community repository (e.g. GitHub). See the Nature Research guidelines for submitting code & software for further information.

Data

Policy information about availability of data

All manuscripts must include a data availability statement. This statement should provide the following information, where applicable:

- Accession codes, unique identifiers, or web links for publicly available datasets
- A list of figures that have associated raw data
- A description of any restrictions on data availability

All the data needed for repeating the experiments described in the paper are available at https://github.com/yluATlzu/3DReconstruction_SE. We have used the following publicly available datasets: EMD-3508 (cryo-EM structure of Escherichia coli 70S ribosome-ArfA-RF2 complex, https://www.emdataresource.org/EMD-3508), EMD-2660 (cryo-EM structure of the Plasmodium falciparum 80S ribosome, https://www.emdataresource.org/EMD-2660), EMPIAR-10028 (Micrographs and particle coordinates of a Plasmodium falciparum 80S ribosome, https://www.ebi.ac.uk/pdbe/emdb/empiar/entry/10028), and EMPIAR-10328 (micrographs and particle coordinates of PTCH1-TI23 complex, https://www.ebi.ac.uk/empiar/EMPIAR-10328/).

Field-specific reporting

Please select the one below that is the best fit for your research. If you are not sure, read the appropriate sections before making your selection.

☒ Life sciences ☐ Behavioural & social sciences ☐ Ecological, evolutionary & environmental sciences

For a reference copy of the document with all sections, see nature.com/documents/nr-reporting-summary-flat.pdf

Life sciences study design

All studies must disclose on these points even when the disclosure is negative.

Sample size	For the simulated datasets, different number of samples (projection images) were tested to show the effects of the sample sizes. For the real datasets, 531 class averages were generated from 11,983 particles selected using the defocus values for EMPIAR-10028, and 390 class averages were generated from 307,652 particles selected using the defocus values for EMPIAR-10328.
Data exclusions	For the real dataset EMPIAR-10028, 136 out of 499 micrographs with defocus values lower than -2.2µm were selected, yielding 11,983 particles.
Replication	The experiments were repeated several times, and yielding similar results.
Randomization	When generating the simulated datasets, Gaussian noises with different random seeds were used in the experiments.
Blinding	Researchers were blinded to the expected results. Different researchers were responsible for data preparation, coding, result collection, and data analysis.

Reporting for specific materials, systems and methods

We require information from authors about some types of materials, experimental systems and methods used in many studies. Here, indicate whether each material, system or method listed is relevant to your study. If you are not sure if a list item applies to your research, read the appropriate section before selecting a response.

Materials & experimental systems

n/a	Involved in the study	
☒	☐	Antibodies
☒	☐	Eukaryotic cell lines
☒	☐	Palaeontology and archaeology
☒	☐	Animals and other organisms
☒	☐	Human research participants
☒	☐	Clinical data
☒	☐	Dual use research of concern

Methods

n/a	Involved in the study	
☒	☐	ChIP-seq
☒	☐	Flow cytometry
☒	☐	MRI-based neuroimaging
